# Uncoupling Insulin Sensitivity From Longevity: A Sex‐Dependent Effect of Hepatic Glucagon Signaling

**DOI:** 10.1111/acel.70349

**Published:** 2026-01-02

**Authors:** Alexander Tate Lasher, Ben Heckman, Parth Sarker, Kaimao Liu, Liou Y. Sun

**Affiliations:** ^1^ Department of Biology University of Alabama at Birmingham Birmingham Alabama USA

## Abstract

Glucagon, a key hormone in maintaining euglycemia during fasting, also exerts broad metabolic effects, including regulation of lipid oxidation, adiposity, insulin sensitivity, and metabolic rate. However, its role in aging and longevity remains largely unexplored, a significant omission given the extensive research on dietary restriction and insulin signaling in lifespan modulation. Here, we investigated the impact of hepatic glucagon receptor (GCGR) signaling on lifespan using a liver‐specific GCGR knockout (LKO) mouse model. While male LKO mice exhibited normal lifespan, female LKO mice displayed a significant reduction in survival. Strikingly, and in contrast to prevailing expectations based on metabolic improvements, this shortened lifespan in females occurred despite marked enhancements in metabolic health, including reduced body weight and adiposity, preferential glucose oxidation, elevated metabolic rate, and enhanced glucose tolerance and insulin sensitivity throughout adulthood. Underpinning this detrimental outcome, transcriptomic and biochemical analyses revealed a striking, female‐specific activation of pro‐inflammatory pathways, notably NF‐κB and cGAS‐STING signaling, in the liver and kidney of aged LKO mice as well as reduced expression of hepatic xenobiotic metabolism genes. These findings identify a novel, sexually dimorphic role for the hepatic glucagon receptor in regulating lifespan, linking its interruption in females to late‐life inflammation and reduced longevity despite an otherwise beneficial metabolic phenotype.

## Graphical abstract

Hepatic glucagon receptor knockout enhances metabolic health but shortens lifespan in female mice due to late‐life inflammation via NF‐κB and cGAS‐STING pathways, revealing a sexually dimorphic role in longevity despite metabolic benefits.

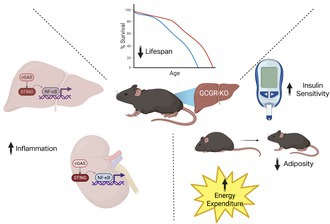

## 1 Introduction

Glucagon, classically considered a counter to insulin action, is a 29 amino acid peptide secreted from the α‐cells within the pancreatic islets of Langerhans. Hypoglycemia induced during fasting or amino acid cues trigger the release of glucagon where it enters systemic circulation (Ohneda et al. 1969; Unger et al. 1969) and elicits its physiological effect by binding the glucagon receptor (GCGR). Canonical glucagon action involves glucagon binding the GCGR, stimulating intracellular cAMP accumulation and subsequent phosphorylation of the cAMP response element binding protein (CREB) (Herzig et al. 2001; Jelinek et al. 1993), and ultimately elevating blood glucose by inducing the transcription of gluconeogenic and glycogenolytic genes (Habegger 2022). Apart from its role in maintaining glycemia between feedings, both agonism (Kim et al. 2018) and deletion (Sørensen et al. 2006) of the GCGR are known to improve insulin sensitivity demonstrated by greater reductions in blood glucose following an insulin challenge. Further, glucagon is also known to acutely elevate energy expenditure in both rodents (Davidson et al. 1960) and humans (Nair 1987), and stimulate hepatic lipolysis (Longuet et al. 2008; Perry et al. 2020). These previous reports indicate that glucagon is a broad and physiologically relevant regulator of fasting metabolism.

Dietary restriction is among the longest studied and most well‐known avenues for extending mammalian lifespan (Swindell 2012). However, despite glucagon's involvement in fasting responses, its action has yet to be rigorously studied in the context of aging. While fasting is known to stimulate glucagon secretion and GCGR activation, impaired glucagon responses have also been observed in extremely long‐lived growth hormone‐deficient mice (Lasher and Sun 2023). This gap in understanding prompted us to directly investigate glucagon's role in aging using liver‐specific GCGR knockout (LKO) mice.

We found that LKO mice exhibited improved metabolic health, including reduced body fat and enhanced insulin sensitivity, traits typically linked to extended lifespan. Unexpectedly, female LKO mice displayed significantly reduced survival, whereas male lifespan remained unaffected. Transcriptomic and western blot analyses revealed female‐specific activation of pro‐inflammatory NFκB and cGAS‐STING pathways and reduced expression of hepatic xenobiotic metabolism genes in aged LKO females, changes absent in males and controls. These findings uncover a sexually dimorphic role for hepatic GCGR signaling in longevity, linking its disruption in females to late‐life inflammation, impaired stress responses, and reduced survival despite what might typically be considered metabolic benefits. Our results challenge the assumption that enhanced insulin sensitivity universally extends lifespan and highlight the need to consider sex‐specific mechanisms in aging research.

## 2 Results

### 2.1 Female Mice Lacking the Hepatic GCGR Are Short Lived

**FIGURE 1:**
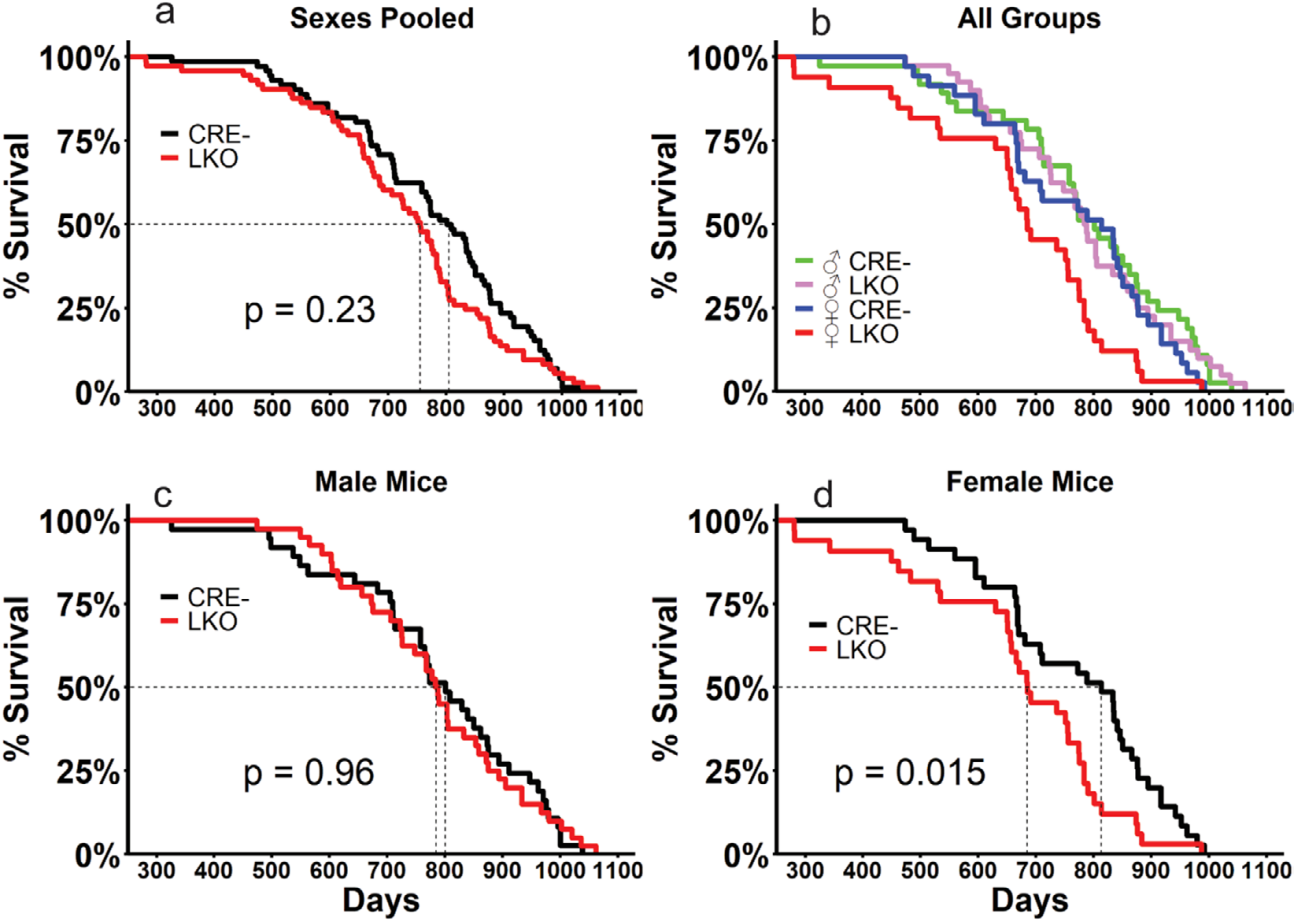
Female mice lacking the hepatic GCGR are short lived. Kaplan–Meier survival curves for male and female mice with functional hepatic GCGR signaling (CRE‐) or with a liver‐specific GCGR knockout (LKO) pooled together (a), all groups assessed (b), male mice (c) and female mice (d). *p*‐values presented represent the effect of genotype as determined by logrank test. Additional statistical analyses of lifespan are provided in Table 1. *n* = 33–40 per group.

We employed a previously detailed mouse model of Cre/LoxP mediated liver‐specific GCGR disruption and validated the specificity of this knockout using tissues known to express the GCGR (Beaudry et al. 2019). Consistent with the original report of this model (Longuet et al. 2013), GCGR expression was only reduced in the liver (Figure S1a,b). To gauge the consequence of interrupted GCGR signaling, we evaluated the lifespan of mice with liver‐specific GCGR deficiency (LKO) and the lifespan of Cre negative (CRE‐) controls. When the sexes were combined, LKO mice displayed no difference in lifespan (CRE‐ median 804 days, LKO median 755 days; Figure 1a). When sexes were analyzed separately, male mice displayed no differences in lifespan (CRE‐ median 801 days, LKO median 785 days, Figure 1b,c) while female LKO mice displayed significantly shorter lifespans (CRE‐ median 813 days, LKO median 685 days—a 19% reduction; Figure 1b,d). While no differences were detected between the sexes in our CRE‐ mice, female LKO mice were also significantly shorter lived compared to male LKO mice (female LKO median 685 days, male LKO 785 days—a 15% reduction, Table 1).

**TABLE 1:** Lifespan analysis of mice.

Sex	Genotype	Median lifespan	Logrank *p*‐value	Cox proportional hazard		Maximal lifespan	
				Hazard ratio	*p*	75th *p*‐value	90th *p*‐value
Pooled	CRE‐ (*n* = 72)	804 days	NS	1	NS	NS	NS
	LKO (*n* = 73)	755 days		1.2202			
Male	CRE‐ (*n* = 37)	801 days	NS	1	NS	NS	NS
	LKO (*n* = 40)	785 days		1.0103			
Female	CRE‐ (*n* = 35)	813 days	0.0150	1	0.0160	0.0247	0.0553
	LKO (*n* = 33)	685 days		1.8391			
Male CRE‐ (*n* = 37)		801 days	NS	1	NS	NS	NS
Female CRE‐ (*n* = 35)		813 days		1.3703			
Male LKO (*n* = 40)		785 days	0.0042	1	0.0051	0.0169	0.0351
Female LKO (*n* = 33)		685 days		2.0019			

Cox proportional hazard analysis of pooled sexes revealed no significant differences in the hazard ratio between CRE‐ and LKO mice when sexes are pooled or when males are analyzed (Table 1). Female LKO mice displayed a significantly greater hazard ratio of 1.8391 compared to female CRE‐ mice (Table 1). No significant difference was observed in the hazard ratio of female CRE‐ mice relative to male CRE‐ mice, the female LKO hazard ratio was significantly greater compared to male LKO mice (Table 1). Quantile regression (Wang et al. 2004) was employed to evaluate survival at the upper percentiles (75th and 90th) of life. Female LKO survival was significantly shorter at the 75th percentile compared to CRE‐ females, and a trend (*p* = 0.0553) was observed for reduced survival at the 90th percentile (Table 1). Female LKO survival at the 75th and 90th percentile was significantly shorter compared to male LKO mice (Table 1). No differences in maximal lifespan were detected for any of the other groups evaluated.

### 2.2 Hepatic GCGR Deficiency Protects Against Age‐Associated Obesity

**FIGURE 2:**
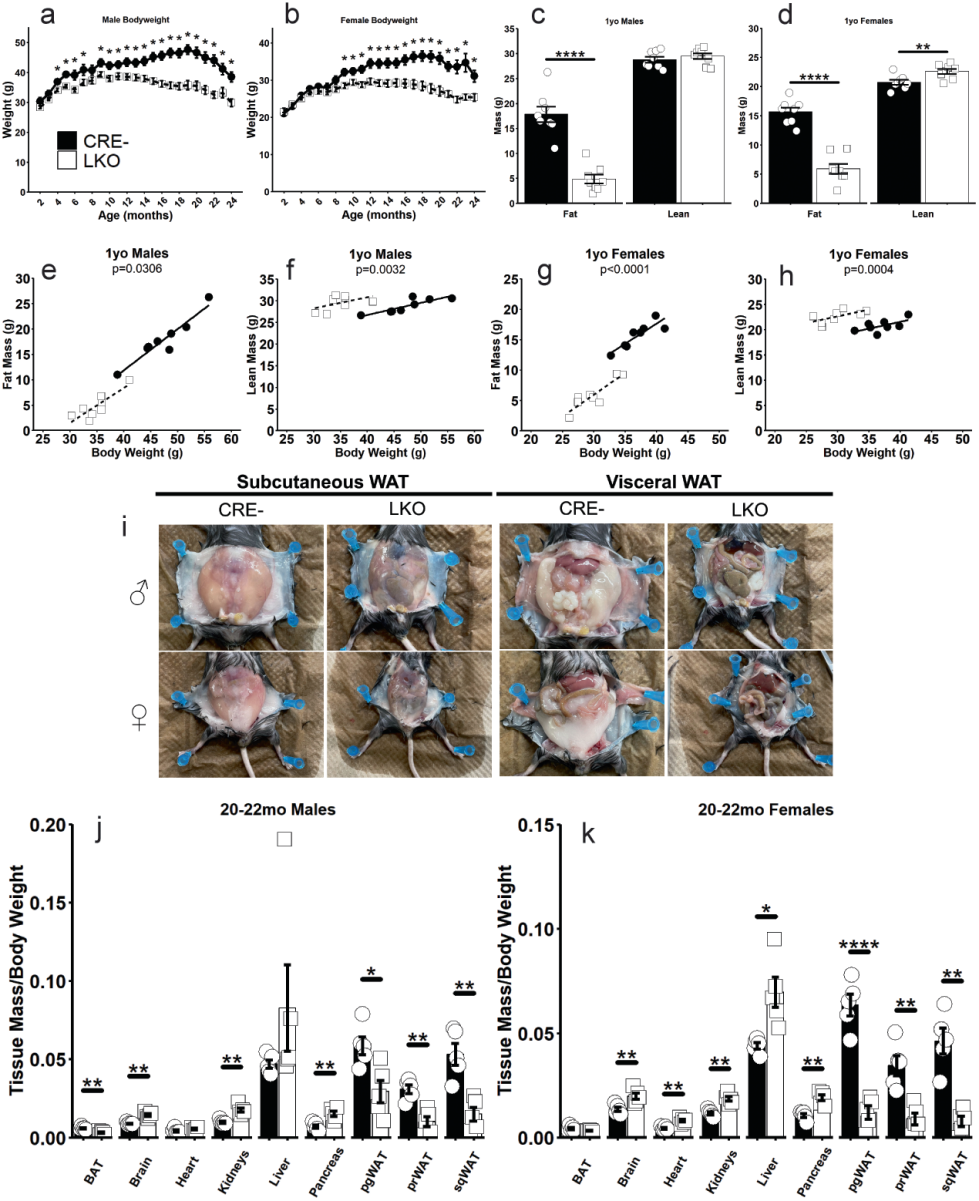
Hepatic GCGR deficiency protects against age‐associated obesity. Longitudinal bodyweights of male (a) and female (b) mice. Fat and lean mass assessed by QMR in 1‐year old male (c) and female (d) mice. Male fat mass (e) and lean mass (f) or female fat mass (g) and lean mass (h) were analyzed with bodyweight as a covariate to control for the allometric effect of reduced body weight on body composition. Representative images of white adipose tissue (WAT) depots observed during dissection (i). Organ tissue mass to body weight ratio recorded during dissections of male (j) and female (k) mice. *p < 0.05, **p < 0.01, ***p < 0.001, ****p < 0.0001 as determined by two‐tailed *t*‐test (c, d, j, k) or by two‐way ANOVA with Tukey HSD post hoc comparisons (a, b). *p*‐values presented (e–h) represent the main effect of genotype after controlling for the effect of bodyweight as determined by ANCOVA. Data presented as mean ± SEM with points representing individual mice.

Body weight was comparable during the first 3 months of age in male mice (Figure 2a) and first 8 months of age in female mice (Figure 2b). Beyond these ages, LKO mice weighed significantly less than their sex‐matched CRE‐ controls. Body composition analysis at 1 year of age revealed significant reductions in body fat mass of male and female LKO mice, as well as significant elevations in female LKO lean mass (Figure 2c,d). Body fat mass and lean mass were analyzed with body weight as a covariate to control for the allometric relationship these compartments display (Tschöp et al. 2011). After accounting for the dramatic differences in body weight at 1 year old, fat mass was significantly reduced, and lean mass was significantly elevated in male and female LKO mice (Figure 2e–h). In advanced age, this difference in body composition was obvious as 21–22‐month‐old LKO mice displayed notably smaller subcutaneous and visceral white adipose depots (Figure 2i). The ratio of all white adipose depot weights to bodyweight were significantly reduced in LKO males and females, as was the male LKO brown adipose depot (Figure 2j,k). The ratio of brain, kidney, and pancreas weight to body weight was significantly elevated in male and female LKO mice, while the heart and liver weights to body weight ratios were also elevated in female LKO mice (Figure 2j,k). Unadjusted organ weights largely followed this pattern (Figure S2a,b). It is also worth noting the damaged and irregular appearance of the female kidneys (Figure S2c) and liver (Figure S2c,d).

### 2.3 Hepatic GCGR Deletion Elevates Glucose Utilization and Insulin Sensitivity

**FIGURE 3:**
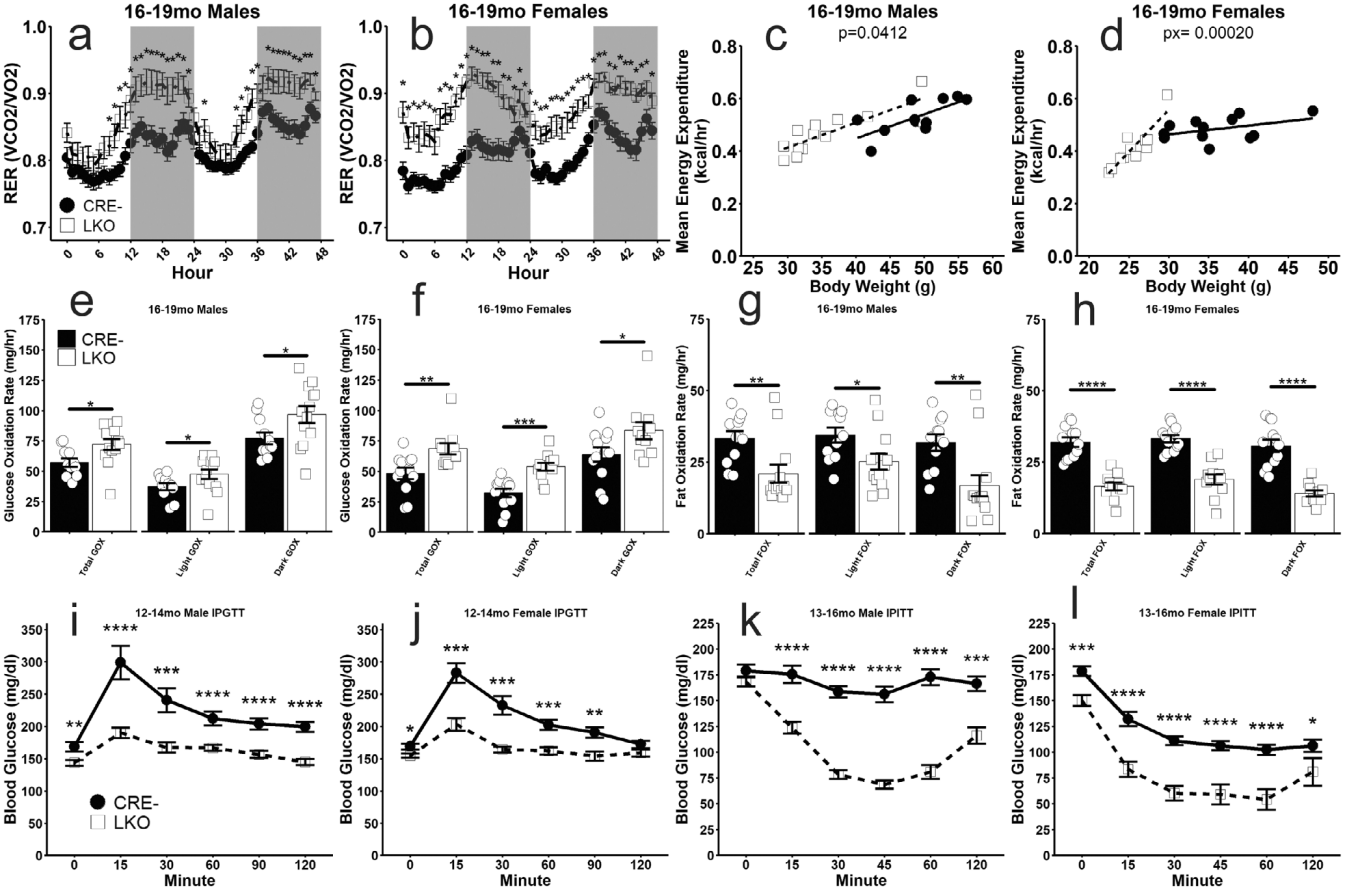
Hepatic GCGR deletion elevates glucose utilization and insulin sensitivity. Respiratory exchange ratio (RER) recorded during indirect calorimetry in male (a) and female (b) mice. Mean energy expenditure calculated during the indirect calorimetry experiment, with bodyweight as a covariate in male (c) and female (d) mice. Mean rates of glucose oxidation (e, f) and fat oxidation (g, h) calculated as detailed in the methods section, for male and female mice as indicated. Intraperitoneal glucose tolerance tests (IPGTT) in overnight fasted male (i) and female (j) mice. IP insulin tolerance tests (IPITT) in non‐fasted male (k) and female (l) mice. *p < 0.05, **p < 0.01, ***p < 0.001, ****p < 0.0001 as determined by two‐tailed *t*‐test (c–h) or by two‐way ANOVA with Tukey HSD post hoc comparisons (a, b, g–j). *p*‐values presented indicate the main effect of genotype, and Px indicates the interaction of genotype and bodyweight as determined by ANCOVA. Data presented as mean ± SEM with points representing individual mice. *N* = 11–24 per group.

Respiratory exchange ratio (RER), calculated during our indirect calorimetry experiments, was significantly elevated in male and female LKO mice (Figure 3a,b), suggesting increased utilization of glucose to meet organismal energy demands (Zurlo et al. 1990). Unadjusted energy expenditure was significantly lower in LKO males and females (Figure S3a,b). After adjusting for the dramatic differences in bodyweight, energy expenditure was elevated in male LKO mice (Figure 3c) and a significant interaction effect of genotype and bodyweight on energy expenditure was detected in female LKO mice (Figure 3d), indicating that increases in bodyweight result in more dramatic elevations in energy expenditure in LKO females compared to CRE‐ females. Ambulatory activity was comparable in male mice, and significantly lower during the dark cycle in female LKO mice (Figure S3c,d). Interestingly, LKO males and females consumed significantly more food compared to CRE‐ mice (Figure S3e,f) suggesting that reduced food consumption does not account for the bodyweight difference observed in our mice. To further probe nutrient utilization in these mice, glucose and fat oxidation (GOX and FOX, respectively) were estimated during our indirect calorimetry experiments according to the work of Simonson and DeFronzo (1990). GOX rate was significantly elevated (Figure 3e,f), and FOX rate was significantly reduced (Figure 3g,h) in LKO mice during light, dark, and the total calorimetry experiment. Intraperitoneal (IP) glucose tolerance testing revealed significantly lower blood glucose at nearly all time points assessed following a glucose challenge in both male and female LKO mice (Figure 3i,j). IP insulin tolerance testing revealed markedly enhanced insulin sensitivity, with blood glucose being lower at all time points assessed following an insulin challenge in male and female LKO mice (Figure 3k,l).

### 2.4 Increased Inflammatory Gene Expression in Female Mice Lacking the Hepatic GCGR

**FIGURE 4:**
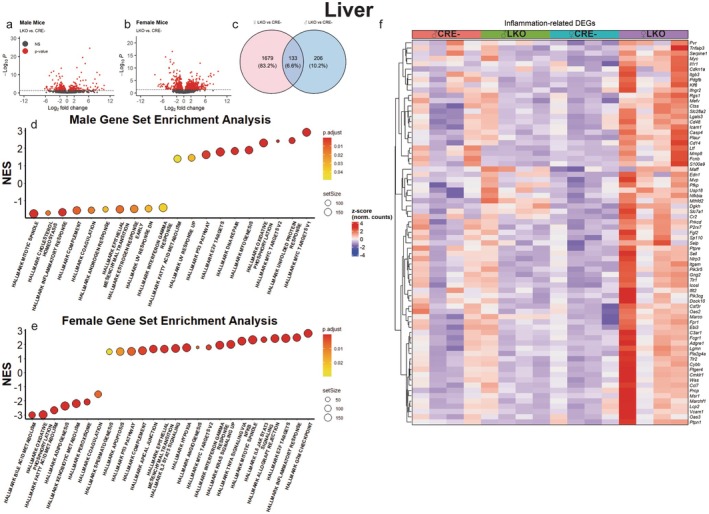
Increased inflammatory gene expression in female mice lacking the hepatic GCGR. Volcano plots of differently expressed genes (DEGs) in livers of 20–22‐month‐old male LKO (a) and female LKO (b) mice relative to sex‐matched CRE‐ mice. Venn diagram of DEGs that are shared (center) and unique (left or right) to female or male LKO mice, relative to sex‐matched controls (c). Normalized enrichment scores (NES) calculated from gene set enrichment analysis of hallmark gene sets curated from the Molecular Signatures Database for male (d) and female (e) mice. Heatmap visualization of DEGs within the hallmark inflammatory response, interferon gamma response, and complement gene sets (termed inflammation‐related), with color scale representing *z*‐scores of normalized gene counts (f). *N* = 4 per group. Fold change was calculated relative to the sex‐matched CRE‐ control group.

We carried out bulk RNA‐sequencing on the livers of 20–22‐month‐old male and female mice to identify potential mechanisms for the differential survival we observed in our colony. Compared to CRE‐ controls, we detected 339 differently expressed genes (DEGs) in male LKO mice (Figure 4a) and 1812 DEGs in female LKO mice (Figure 4b). Of these DEGs, 206 were unique to the male LKOs, 1679 were unique to the female LKOs, and only 133 were shared between male and female LKOs (Figure 4c) indicating striking sex‐specific differences. Gene set enrichment analysis, querying the hallmark gene sets curated from the Molecular Signatures Database (Castanza et al. 2023), revealed 10 upregulated and 10 downregulated hallmark gene sets in male LKO mice (Figure 4d), and 19 upregulated and 7 downregulated gene sets in female LKO mice (Figure 4e). Inspection of these results revealed that the hallmark inflammatory response, interferon gamma response, complement, and oxidative phosphorylation were oppositely regulated in LKO males and females. Sixteen DEGs were observed in the oxidative‐phosphorylation gene set (Figure S4a) and 73 DEGs were observed in the inflammation‐related gene sets (inflammatory response, interferon gamma response, complement; Figure 4f), indicating a unique inflammatory activation in the female LKO mice. Consistent with this, gene ontology biological process overrepresentation analysis in clusters of upregulated DEGs detected multiple inflammatory processes associated with the female LKO transcriptome, while this was generally absent in the male mice (Figure S4b,c). Interestingly, fibroblast growth factor‐21 expression, a hormone whose overexpression has previously been associated with extreme longevity (Y. Zhang et al. 2012), was markedly elevated in both male and female LKO livers (Figure S4d,e). It is also noteworthy that hallmark spermatogenesis was elevated in female LKO mice (Figure 4e). Further inspection of DEGs within this gene set revealed this was driven by increased expression of genes involved in chromosome segregation (Figure S4f), potentially reflecting immune activation in this group (van den Brink et al. 2023).

### 2.5 NFκB and cGAS‐STING Signaling Is Activated in Female Mice Lacking the Hepatic GCGR

**FIGURE 5:**
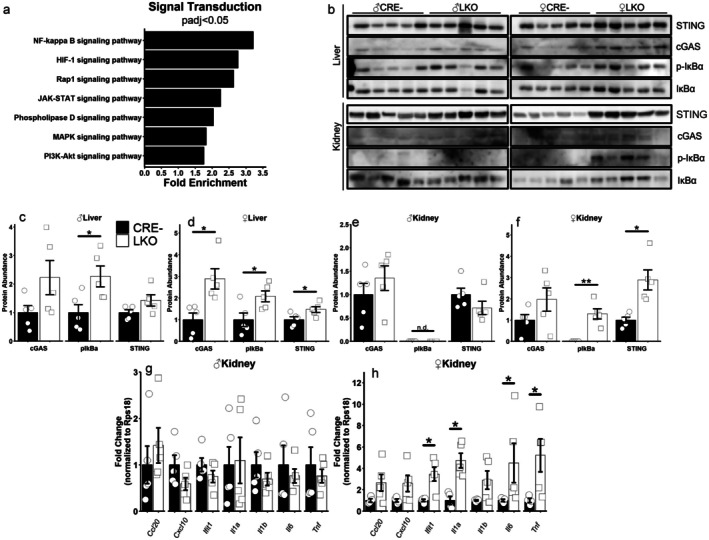
NFκB and cGAS‐STING signaling is activated in female mice lacking the hepatic GCGR. Significantly enriched terms from female DEGs detected in overrepresentation analysis of the KEGG database, with the analysis restricted to the signal transduction subcategory (a). Western blot analysis of STING, cGAS, phospho‐IκBα (Ser32), and total IκBα in the liver and kidney (b). Quantification of the indicated protein abundances in 20–22‐month‐old male (c) and female (d) livers or male (e) and female (f) kidneys. Signal intensities were normalized to total protein stain or unphosphorylated protein. Relative abundance of pro‐inflammatory mRNA transcripts, assessed by RT‐qPCR, in the kidney of 20–22‐month‐old male (g) or female (h) mice, with *Rps18* used as an endogenous control. *p < 0.05; **p < 0.01 as determined by two‐tailed t‐test (c–f) or by Mann–Whitney *U*‐test (g, h). Data presented as mean ± SEM with points representing individual mice. *N* = 4–5 per group.

To elucidate mechanisms driving the inflammatory gene expression in female liver‐specific glucagon receptor knockout (LKO) mice, we performed overrepresentation analysis of upregulated differentially expressed genes (DEGs) using the Kyoto Encyclopedia of Genes and Genomes (KEGG) database, focusing on signal transduction pathways. The NFκB signaling pathway emerged as the most enriched (Figure 5a). Western blot analysis confirmed elevated levels of STING, cGAS, and phosphorylated IκBα—key activators of NFκB signaling implicated in age‐related diseases—in female LKO livers, with increased phospho‐IκBα also observed in male LKO livers (Figure 5b–d). In female LKO kidneys, STING and phospho‐IκBα were significantly elevated, consistent with their inflamed appearance at dissection (Figures 5b,e,f and S2c). RT‐qPCR further revealed increased expression of immune‐related genes in female LKO kidneys, a pattern absent in males and in visceral white adipose tissue (Figures 5g,h and S5a,b). These findings highlight a female‐specific inflammatory response linked to hepatic GCGR disruption, potentially contributing to reduced lifespan.

### 2.6 Reduced Xenobiotic Metabolism Gene Expression in Female Mice Lacking the Hepatic GCGR

**FIGURE 6:**
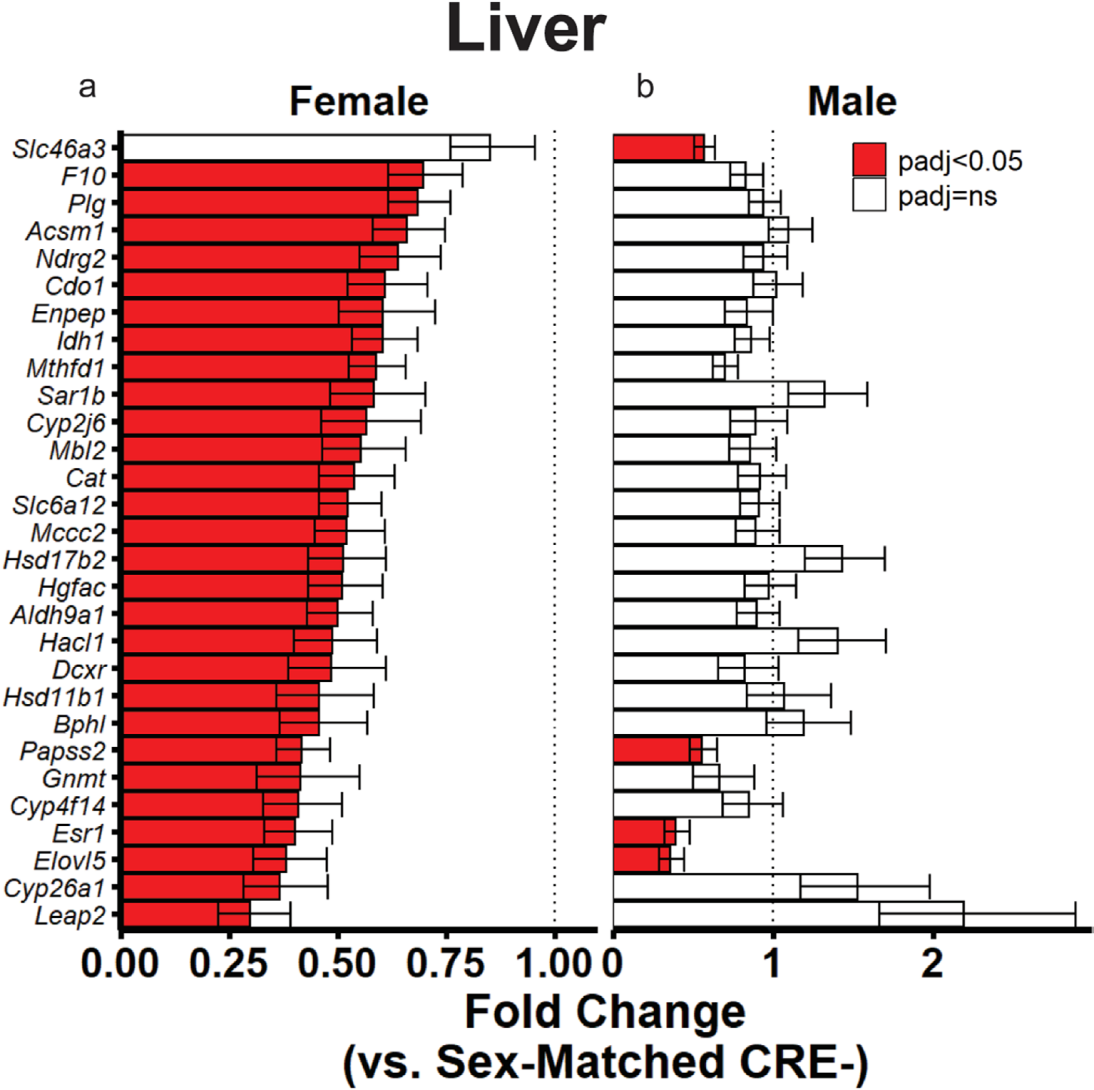
Xenobiotic metabolism gene expression is reduced in female mice lacking the hepatic GCGR. Significantly differently expressed genes related to xenobiotic metabolism in female (a) or male (b) LKO mice relative to sex‐matched CRE‐controls, calculated from DESeq2. Data presented as mean fold change ± SEM. *N* = 4 per group.

Interestingly, we also detected a significantly reduced hallmark xenobiotic metabolism gene set in our female LKO mice (Figure 4e). As changes in this gene set were not detected in our male mice and elevated xenobiotic metabolism has been observed in mice with extended lifespans (Li et al. 2013; Steinbaugh et al. 2012), we further investigated this pathway. Twenty‐nine differently expressed genes within the xenobiotic metabolism gene set were detected in our RNA‐seq dataset. 28 of these were significantly reduced in our female LKO livers (Figure 6a) while only 4 were significantly reduced in male LKO livers (Figure 6b) relative to sex‐matched CRE‐ controls. Together with the data presented in Figure 4d,e, these indicate a clear pattern of reduced xenobiotic metabolism gene expression in the short‐lived female LKO mice.

## 3 Discussion

Genetic manipulation of endocrine pathways has been pivotal in unraveling the mechanisms governing aging. Here, we show that our LKO mice exhibit a striking sexual dimorphism in lifespan: females have significantly reduced survival, whereas males are unaffected. Despite metabolic improvements in adulthood—including reduced body weight and adiposity, increased glucose oxidation, elevated metabolic rate, and enhanced insulin sensitivity—female LKO mice displayed shortened lifespans. Transcriptomic and protein analysis in female LKO mice revealed pronounced NFκB and cGAS‐STING activation and reduced xenobiotic metabolism gene expression, the upregulation of which is typically associated with extended longevity (Li et al. 2013; McElwee et al. 2004; Steinbaugh et al. 2012). This challenges the assumption that metabolic benefits uniformly extend longevity and highlights the need for sex‐specific perspectives in aging research.

Our longevity analysis showed female LKO mice have significantly reduced survival, whereas male lifespans remain unchanged. Notably, the median lifespan of control mice (804 days; 801 days for males, 813 days for females) differs from our prior reports (738 days; 653 days for males, 862 days for females (Adkins‐Jablonsky et al. 2024) or 786 days; 716 days for males, 864 days for females (Lasher et al. 2024)). This potentially reflects the loss of early‐life female survival advantage on a C57BL/6J background versus outbred mice (Cheng et al. 2019). Female LKO mice also displayed reduced maximal survival at the 75th percentile of life (although 90th percentile survival did not reach statistical significance) while no change was observed in males. This contrasts with long‐lived models such as growth hormone mutant mice (Masternak et al. 2009; Wiesenborn et al. 2014; F. Zhang et al. 2020), *Fgf21* transgenic mice (Gliniak et al. 2025; Y. Zhang et al. 2012), and those under long‐term caloric (Barzilai et al. 1998) or methionine restriction (Miller et al. 2005; Nagarajan et al. 2024), where metabolic “improvements”, especially enhanced insulin sensitivity, often correlate with extended lifespan. Our findings align with a past report by Nelson et al. (2012) that increased insulin sensitivity does not universally promote longevity and may even shorten it in mice. These results challenge the notion that metabolic benefits inherently extend lifespan and highlight the critical role of glucagon action in sex‐specific mechanisms of aging research.

Previous studies reported that whole‐body GCGR‐KO mice exhibit significantly reduced lifespan in both sexes (Yu et al. 2012). As the GCGR is predominantly expressed in the liver (Campos et al. 1994), these findings contrast with our observation of reduced lifespan only in female LKO mice. The whole‐body knockout model employed in this previous report showed off‐target effects, including a 10‐fold increase in plasma glucagon in control littermates compared to wild‐type mice from unrelated breeders (Parker et al. 2002), potentially confounding lifespan outcomes. However, a recent report by Bruner et al., using a model free of this issue (Gelling et al. 2003), also reported reduced lifespan in male GCGR‐KO mice (Bruner et al. 2025), suggesting other factors at play. We propose that residual glucagon receptor activity in non‐hepatic tissues in our LKO model may account for the sex‐specific lifespan differences, highlighting the nuanced role of tissue‐specific glucagon signaling in aging.

To investigate the differential survival in LKO mice, we examined hepatic and extrahepatic inflammatory profiles, revealing pronounced upregulation of NFκB signaling in short‐lived female LKO mice, absent in males. This aligns with prior studies linking NFκB activation to reduced lifespan and its inhibition to extended longevity in mice. Hypothalamic NFκB modulation has been shown to shorten or extend lifespan (G. Zhang et al. 2013), while reduced NFκB signaling characterizes long‐lived mutant mice and lifespan‐extending interventions (Elmansi et al. 2024), with the converse observed in lifespan‐shortening conditions (L. Y. Sun et al. 2017). Elevated cGAS and STING protein levels in female LKO liver and kidney suggest a mechanism for NFκB activation, as cGAS‐STING signaling drives NFκB activity (Balka et al. 2020) and is implicated in sex‐dependent progression of age‐related diseases (Khedr et al. 2024). These findings indicate that hepatic GCGR loss triggers female‐specific cGAS‐STING and NFκB‐mediated systemic inflammation, contributing to reduced lifespan.

Additionally, female LKO mice exhibited reduced hepatic xenobiotic metabolism gene expression, a feature absent in males. Such genes, notably cytochrome P450s, are consistently upregulated in long‐lived *C. elegans* (McElwee et al. 2004) and mouse models (Steinbaugh et al. 2012; L. Y. Sun et al. 2013), and are correlated positively with lifespan (Swindell 2007). Their suppression in female LKO mice may potentially contribute to their shortened lifespan, though pinpointing specific genes within this pathway potentially controlling lifespan remains challenging due to their diverse roles. Intriguingly, despite elevated *Fgf21* expression in both male and female LKO mice, no lifespan extension was observed, contrasting with the extended longevity in *Fgf21*‐overexpressing transgenic mice (Y. Zhang et al. 2012), particularly females. This suggests that FGF21's lifespan‐extending effects may require intact hepatic glucagon signaling.

In interpreting our data, it is important to consider that our experimental mice lacked the GCGR throughout development as well as adult life. As a result, it is unclear if these differences in lifespan we observe are the result of hepatic GCGR deficiency during a critical developmental window, or if this is the cumulative effect of lifelong hepatic GCGR inhibition. Previous research has shown that early life nutrient restriction by litter crowding before weaning is sufficient to extend lifespan (L. Sun et al. 2009), and early life growth hormone replacement during the first weeks of life is sufficient to reduce the longevity of long‐lived dwarf mice to that of WTs (Panici et al. 2010; L. Y. Sun et al. 2017). These findings demonstrate that critical developmental windows exist which can dramatically alter the aging process. This presents a key limitation in our model as the GCGR's impact during these windows can't be assessed. Future studies temporally controlling GCGR deletion are warranted to dissect the impact of lifelong versus adulthood GCGR interruption.

## 4 Methods

### 4.1 Mice

Male and female mice with a liver specific deletion of the hepatic glucagon receptor have been previously described (Longuet et al. 2013) and were acquired from the laboratory of Dr. Daniel J. Drucker through a standard material transfer agreement. Briefly, mutant mice with LoxP sites surrounding exons 6–12 of the *Gcgr* (“floxed‐*Gcgr*” mice) were mated with Albumin‐Cre mice (Jackson Labs Strain #003574) to produce floxed‐*Gcgr* mice either expressing or not expressing the Albumin‐Cre transgene. Floxed‐*Gcgr* mice expressing Albumin‐Cre lack the hepatic glucagon receptor and were termed LKO mice. Floxed‐*Gcgr* mice without Albumin‐Cre retain functional glucagon receptors and were termed CRE‐ mice. All mice were maintained on a C57BL/6J genetic background.

Mice were weaned from their mothers at 21 days old, separated by sex, and group housed at a density of 5–7 individuals per cage in a specific pathogen‐free facility maintained on a standard 12‐h light and 12‐h dark cycle at 20°C–23°C. Mice had ad‐libitum access to standard drinking water and rodent chow (NIH‐31 rat and mouse diet, 18% protein, 4% fat) except where mice were fasted before experiments as indicated in figure legends. A separate cohort was used to assess lifespan, where mice were experimentally naive except for regular bodyweight assessments. All animals were housed in facilities accredited by the American Association for the Accreditation of Laboratory Animal Care at the University of Alabama at Birmingham. All procedures involving live animals were approved by the Institutional Animal Care and Use Committee of the University of Alabama at Birmingham.

### 4.2 Lifespan Analysis

Animal health was inspected at a minimum of 3 times per week, at which point deaths were recorded. Mice that were moribund in appearance and unlikely to survive until the next health check, as determined by University of Alabama at Birmingham veterinary care staff unaffiliated with our laboratory and blinded to our experimental design, were euthanized within 24 h of this observation and this time point was considered the death of the animal. Only natural deaths or mice euthanized for a moribund appearance were included in this lifespan analysis. Overall survival was compared between groups using the log‐rank test and Cox proportional hazard models. Maximal lifespan was evaluated using the quantile regression method (Wang et al. 2004) to compare the proportion of surviving mice at the 75th and 90th percentiles of colony survival.

### 4.3 Body Composition Analysis

Mouse bodyweights were recorded 4 times per month on average throughout the duration of this study. Body composition was assessed by quantitative magnetic resonance using an EchoMRI body composition analyzer (EchoMRI, Houston, TX). Data collected included body fat mass, lean mass, and total body weight. To account for differences in bodyweight between groups, fat mass and lean mass were analyzed with bodyweight as a covariate to account for the allometric relationship between these compartments and a mouse's total body weight.

### 4.4 Indirect Calorimetry

Nutrient utilization and metabolic rate were assessed using the comprehensive lab animal monitoring system (CLAMS; Columbus Instruments, Columbus, OH) indirect calorimetry chambers. This system continuously collects oxygen consumption (VO_2_) and carbon dioxide production (VCO_2_) data every 9 min for each individually housed mouse. This system also monitors mouse activity and food consumption by detecting beam breaks in an infrared laser grid within each chamber and food weight changes in the feeder‐balance systems of each chamber. For food consumption, only periods where the feeder‐balance system did not register gains were considered (such as when the mouse sits on the feeder). This was done to avoid quantitating the mouse's body weight as food consumed, however it should be noted that this also ignores food that is consumed if the mouse happens to feed while seated in the feeder.

Mice were moved from group housing in their home cage to individual housing for 1 week prior to calorimetry studies to allow for acclimation to individual housing. Following this period, mice were individually housed in CLAMS cages for approximately 72 h. Only calorimetry data collected during the final 48 h was considered in our analysis, as the first 24 h was considered an additional acclimation period to allow the mouse to adjust to the CLAMS environment. Respiratory exchange ratio (RER) was calculated as VCO_2_/VO_2_. Rates of glucose oxidation and fat oxidation were calculated as 4.57(VCO_2_)–3.23(VO_2_) and 1.69(VO_2_)–1.69(VCO_2_), respectively, as detailed by Simonson and DeFronzo (1990). Energy expenditure was calculated as VO_2_(3.815 + 1.232(VCO_2_/VO_2_)) according to the work of Graham Lusk (1928). Body weight was used as a covariate in the analysis of energy expenditure.

### 4.5 Glucose and Insulin Tolerance Testing

Mice were fasted overnight prior to glucose tolerance tests (GTT). Insulin tolerance testing (ITT) was carried out in ad‐lib fed mice, with the food removed approximately 15 min prior to testing, in an effort to avoid the hypoglycemic events we observed in our LKO mice during pilot experiments. For GTTs, mice were injected intraperitoneally (IP) with 1 g/kg glucose prepared in normal saline. For ITTs, mice were injected IP with 1 U/kg insulin (Humulin‐R, Eli‐Lilly) prepared in normal saline. Blood glucose was measured from a tail nick using a PRESTO handheld glucometer (AgaMatrix, Salem, NH) immediately prior to injections (“minute 0”) and at the time points following injection as indicated.

### 4.6 RNA Sequencing

Tissues were dissected from 20 to 22‐month‐old mice following CO_2_‐induced deep anesthesia and cervical dislocation. Tissues were rapidly frozen on dry ice and maintained at −80°C until analysis. RNA was isolated from frozen livers using the RNeasy plus mini kit (Qiagen, Hilden, Germany) following the manufacturer protocol and submitted for 150 bp paired‐end sequencing at a depth of 30 M reads on the NovaSeq X Series platform. RNA quality assurance, poly‐A selection, library preparation, and sequencing was carried out by Novogene (Sacramento, CA). A mouse genome (GRCm39) index was generated using STAR (v 2.7.1a) with the option —*sjdbOverhang 149*. Raw reads were aligned to the mouse genome using this index with STAR (v 2.7.1a) and the following options: —*outFilterType BySJout* —*outFilterMultimapNmax 20* —*alignSJoverhangMin 8* —*alignSJDBoverhangMin 1* —*outFilterMismatchNmax 999* —*alignIntronMin 20* —*alignIntronMax 1000000* —*alignMatesGapMax 1000000*. Gene level read counting was carried out using subread (v2.0.6) featureCounts with the following options: ‐*p*‐*s 2*. Differential gene expression analysis was carried out using the DESeq2 (v1.46.0) in R (v4.4.2) with the Wald test used for comparisons. Gene set enrichment analysis and overrepresentation analysis were carried out using clusterProfiler (v4.14.4) in R. For gene set enrichment analysis, the Wald test statistic calculated from DESeq2 was used to rank genes. The differentially expressed genes detected in male and female mice are available in Tables S1 and S2, respectively. Raw sequencing reads have been deposited at the Gene Expression Omnibus (accession GSE302496) and will be made publicly available at the time of publication.

### 4.7 Western Blotting

Frozen liver and kidney tissues were homogenized in ice cold RIPA buffer (50 mM Tris–HCl pH 8.0, 1 mM EDTA, 1% Triton X‐100, 0.1% SDS, 150 mM NaCl) supplemented with protease and phosphatase inhibitor cocktail (Cell Signaling Technology, Danvers, MA catalog #5872). Homogenates were incubated on ice with agitation for 30 min, sonicated, and centrifuged for 10 min at 12,000 × *g* at 4°C. The supernatant was collected, and protein concentration was determined using the DC assay (Bio‐Rad, Hercules, CA catalog #5000112). Equivalent amounts of total protein were loaded into Laemmli loading buffer (Bio‐Rad, Hercules, CA #1610747) supplemented with 2‐mercaptoethanol, heated for 10 min at 95°C, and separated by SDS‐PAGE. Proteins were transferred to PVDF membranes, which were stained with ponceau red to verify transfer success, and then blocked for 30 min with 5% bovine serum albumin in tris‐buffered saline (20 mM Tris pH 7.6, 150 mM NaCl) with 0.1% Tween‐20 (TBST). Membranes were probed with primary antibodies diluted in blocking buffer against the target protein overnight at 4°C, washed in TBST, probed with HRP‐conjugated secondary antibodies raised against the host species of the primary antibody, washed again in TBST, then imaged using SuperSignal West Pico PLUS Chemiluminescent Substrate (Thermo Fisher catalog #34580) or SuperSignal West Femto Maximum Sensitivity Substrate (Thermo Fisher catalog #34094). Membranes were imaged using a PXi imager (Syngene, Cambridge, UK). Band intensities were normalized to total protein stain intensity and quantified using NIH ImageJ software. Antibody information is available in Table S3.

### 4.8 RT‐qPCR

RNA was extracted from frozen livers as described above, or from frozen kidney and white adipose tissue using TRIzol (Invitrogen, Carlsbad CA) according to the manufacturer's protocol. Total RNA was reverse transcribed using M‐MuLV Reverse Transcriptase (New England Biolabs, Ipswich, MA catalog #M0253) following manufacturer recommendations. Quantitative PCR was carried out using a QuantStudio 3 system (Applied Biosystems) with Luna Universal QPCR Master Mix (New England Biolabs, Ipswich, MA catalog #M3003) at a final concentration of 1× in a 10 μL reaction volume. Relative gene expression was assessed using the 2^−ddCt^ method with *Rps18* used as an endogenous control. Primer sequences used are available in Table S4.

### 4.9 Statistical Analysis

Lifespan and RNA‐sequencing data were analyzed as detailed above. Two group means were compared using the two‐tailed *t*‐test or the Mann–Whitney *U*‐test as indicated in figure legends; where data were taken over time, groups were compared using a two‐way repeated measure ANOVA with Tukey HSD post hoc testing carried out where significant effects were observed; where a covariate was considered, groups were compared using ANCOVA. For all analyses, a *p*‐value of less than 0.05 (after adjusting for multiple comparisons, where appropriate) was considered statistically significant. All statistical analysis and figure generation was carried out using the R programming language.

## Author Contributions

L.Y.S. conceptualized the study. L.Y.S. overall direction and secured funding. A.T.L., B.H., P.S., and K.L. designed experiments, conducted experiments, and collected data. A.T.L. and L.Y.S. analyzed data. A.T.L. drafted the original manuscript. All authors edited the manuscript and provided critical feedback that helped shape the final form of this report.

## Funding

The research was supported by NIH grant AG085793 to L.Y.S. The UAB Small Animal Phenotyping Core is supported by NIH grant P30DK056336 (to the UAB Nutrition and Obesity Research Center), by NIH grant P30DK079626 (to the UAB Diabetes Research Center), and by NIH grant P30AG050886 (to the UAB Nathan Shock Center). A.T.L. is supported by NIH grant T32HD071866.

## Conflicts of Interest

The authors declare no conflicts of interest.

## Supporting information


**Figure S1:** Relative expression of glucagon receptor mRNA transcripts in the indicated tissues for male (a) and female (b) 20–22‐month‐old mice. *p < 0.05 as determined by Mann–Whitney *U*‐test. Data presented as mean ± SEM with points representing individual mice. *N* = 4–5 per group.
**Figure S2:**. Unadjusted organ masses in 20–22‐month‐old male (a) and female (b) mice as indicated. Representative gross morphology of liver and kidney in 20–22‐month‐old male and females as indicated (c). Representative hematoxylin and eosin stained liver sections, with scale bar representing 100 μm (d). *p < 0.05 as determined by two‐tailed *t*‐test. Data presented as mean ± SEM with points representing individual mice. *N* = 5 per group.
**Figure S3:**. Bodyweight adjusted energy expenditure in male (a) and female (b) mice calculated during indirect calorimetry. Ambulatory activity, determined by the number of infrared beam breaks recorded for each mouse, calculated during indirect calorimetry for males (c) and females (d). Mean hourly food consumption for male (e) and female (f) mice recorded during indirect calorimetry. *p < 0.05, **p < 0.01, as determined by two‐way ANOVA with Tukey HSD post hoc comparisons (a–d) or by two‐tailed *t*‐test (e, f). Data presented as mean ± SEM with points representing individual mice. *N* = 11–13 per group.
**Figure S4:** Heat map visualization of differentially expressed genes (DEGs) in the oxidative‐phosphorylation gene set (a). Heat map k‐means clustering of DEGs in male LKO mice (b) and female LKO mice (c), relative to sex‐matched controls. The top 3 over‐represented gene ontology biological process terms associated with each cluster are presented to the right (b, c). Fold change in liver *Fgf21* expression, reproduced from the DESeq2 output with the log2 transformation removed from the fold change values in male (d) and female (e) mice. DEGs in the Hallmark Spermatogenesis gene set, with top 5 over‐represented gene ontology biological process terms associated with these genes (f). Padj values represent the adjusted *p*‐values from the DESeq2 output. Color scales represent *z*‐scores of normalized counts. *N* = 4/group.
**Figure S5:**. White adipose gene expression. Relative gene expression in perigonadal visceral white adipose tissue (vWAT) in male (a) and female (b) mice with *Rps18* used as an endogenous control. **p < 0.01 as determined by two‐tailed *t*‐test. Data presented as mean ± SEM with points representing individual mice. *N* = 5 per group.

**Table S1:** Differently expressed genes detected in male livers during RNA‐seq data analysis.

**Table S2:** Differently expressed genes detected in female livers during RNA‐seq data analysis.

**Table S3:** Antibodies used in western blotting experiments.

**Table S4:** Primer sequences used.

## Data Availability

RNA‐sequencing data has been deposited at the GEO with accession number GSE302496 and will be made publicly available at the time of publication. The data supporting the findings of this study are available from the corresponding author upon reasonable request and will be made available as Supporting Information at the time of publication.
