# Supplementary material for: Uncoupling Insulin Sensitivity From Longevity: A Sex‐Dependent Effect of Hepatic Glucagon Signaling
Source: Aging Cell. 2026 Jan 2;25(1):e70349. doi: 10.1111/acel.70349 (PMC12759044; doi:10.1111/acel.70349)
Supplement: Supplementary file 1 — Figure S1: Relative expression of glucagon receptor mRNA transcripts in the indicated tissues for male (a) and female (b) 20–22‐month‐old mice. *p < 0.05 as determined by Mann–Whitney U‐test. Data presented as mean ± SEM with points representing individual mice. N = 4–5 per group. Figure S2:. Unadjusted organ masses in 20–22‐month‐old male (a) and female (b) mice as indicated. Representative gross morphology of liver and kidney in 20–22‐month‐old male and females as indicated (c). Representative hematoxylin and eosin stained liver sections, with scale bar representing 100 μm (d). *p < 0.05 as determined by two‐tailed t‐test. Data presented as mean ± SEM with points representing individual mice. N = 5 per group. Figure S3:. Bodyweight adjusted energy expenditure in male (a) and female (b) mice calculated during indirect calorimetry. Ambulatory activity, determined by the number of infrared beam breaks recorded for each mouse, calculated during indirect calorimetry for males (c) and females (d). Mean hourly food consumption for male (e) and female (f) mice recorded during indirect calorimetry. *p < 0.05, **p < 0.01, as determined by two‐way ANOVA with Tukey HSD post hoc comparisons (a–d) or by two‐tailed t‐test (e, f). Data presented as mean ± SEM with points representing individual mice. N = 11–13 per group. Figure S4: Heat map visualization of differentially expressed genes (DEGs) in the oxidative‐phosphorylation gene set (a). Heat map k‐means clustering of DEGs in male LKO mice (b) and female LKO mice (c), relative to sex‐matched controls. The top 3 over‐represented gene ontology biological process terms associated with each cluster are presented to the right (b, c). Fold change in liver Fgf21 expression, reproduced from the DESeq2 output with the log2 transformation removed from the fold change values in male (d) and female (e) mice. DEGs in the Hallmark Spermatogenesis gene set, with top 5 over‐represented gene ontology biological process terms associated with th [file ACEL-25-e70349-s004.docx]

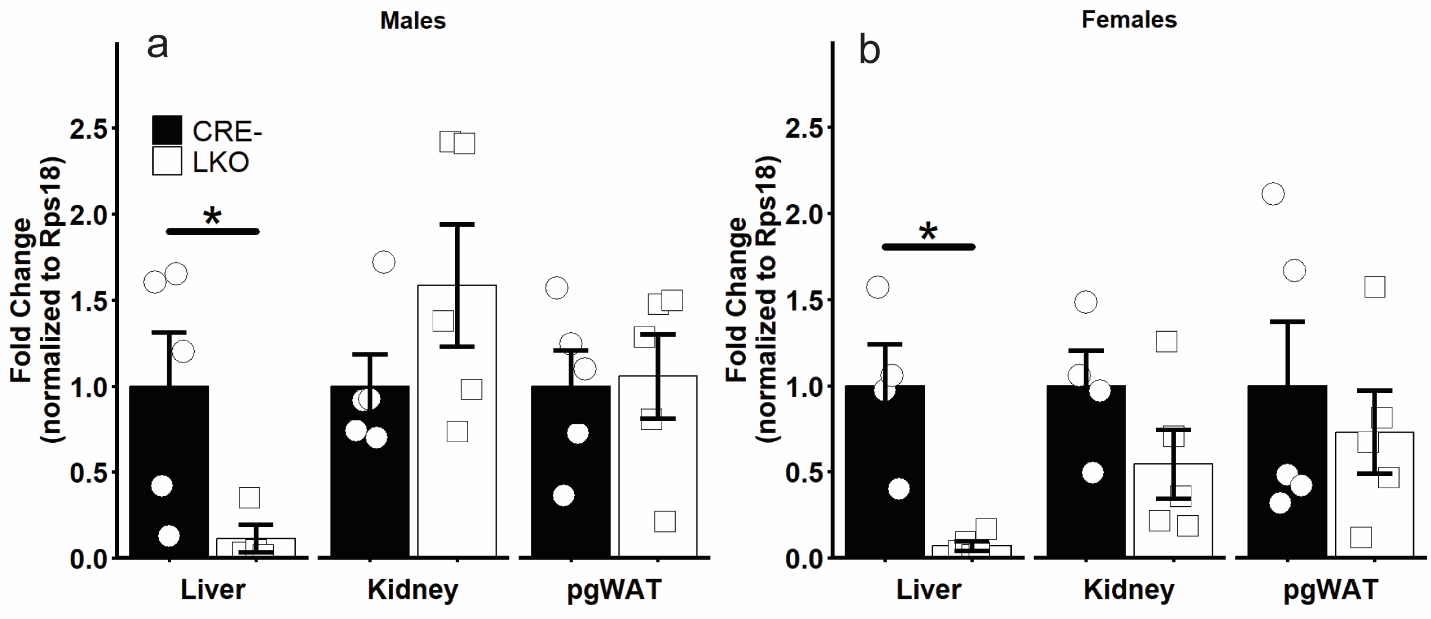


**Supplemental Figure S1.** Relative expression of glucagon receptor mRNA transcripts in the indicated tissues for male (a) and female (b) 20-22-month-old mice. *p<0.05 as determined by Mann-Whitney U-test. Data presented as mean ± SEM with points representing individual mice. N=4-5 per group.


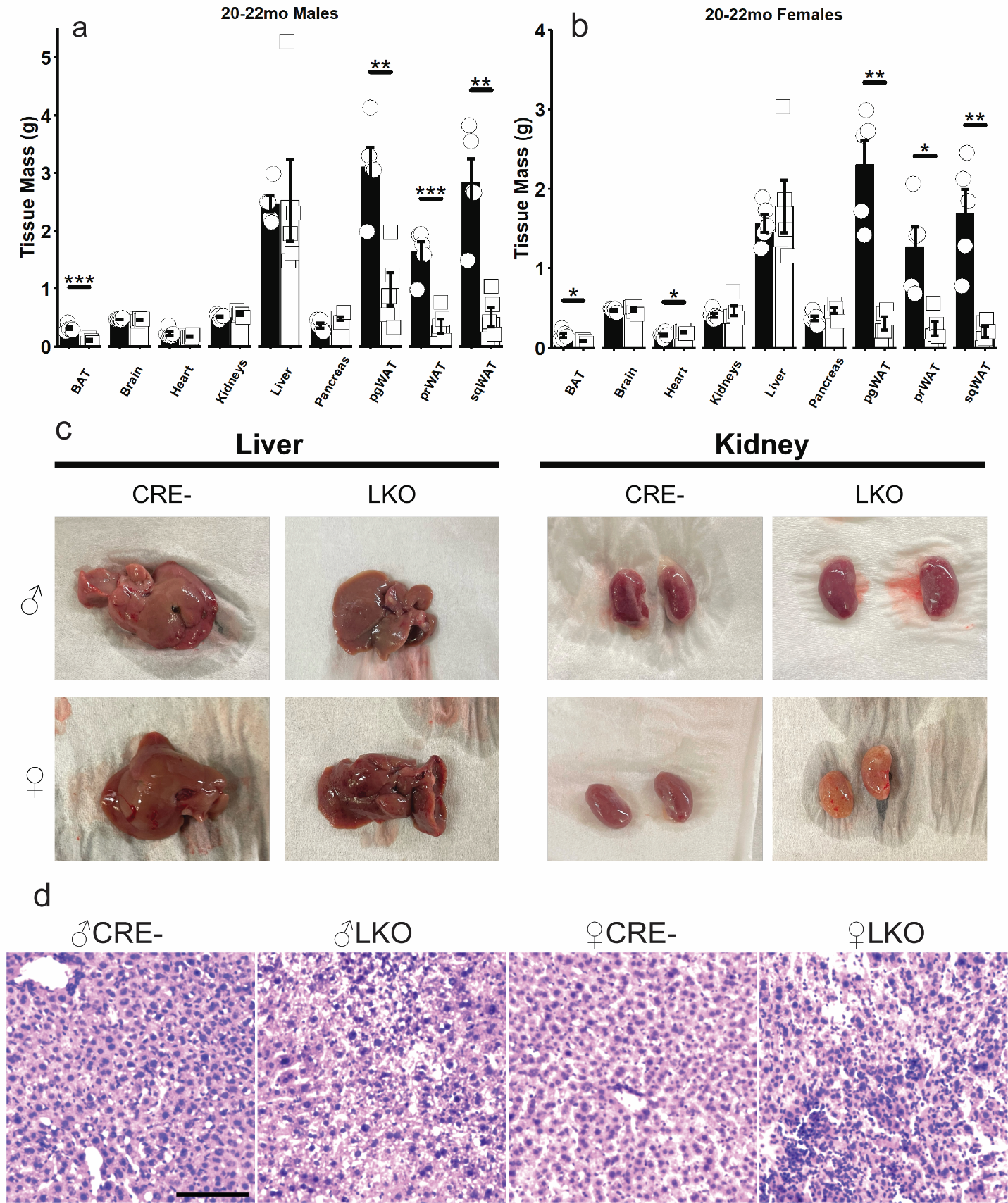


**Supplemental Figure S2**. Unadjusted organ masses in 20-22-month-old male (a) and female (b) mice as indicated. Representative gross morphology of liver and kidney in 20-22-month-old male and females as indicated (c). Representative hematoxylin and eosin stained liver sections, with scale bar representing 100µm (d). *p<0.05 as determined by two-tailed t-test. Data presented as mean ± SEM with points representing individual mice. N=5 per group.


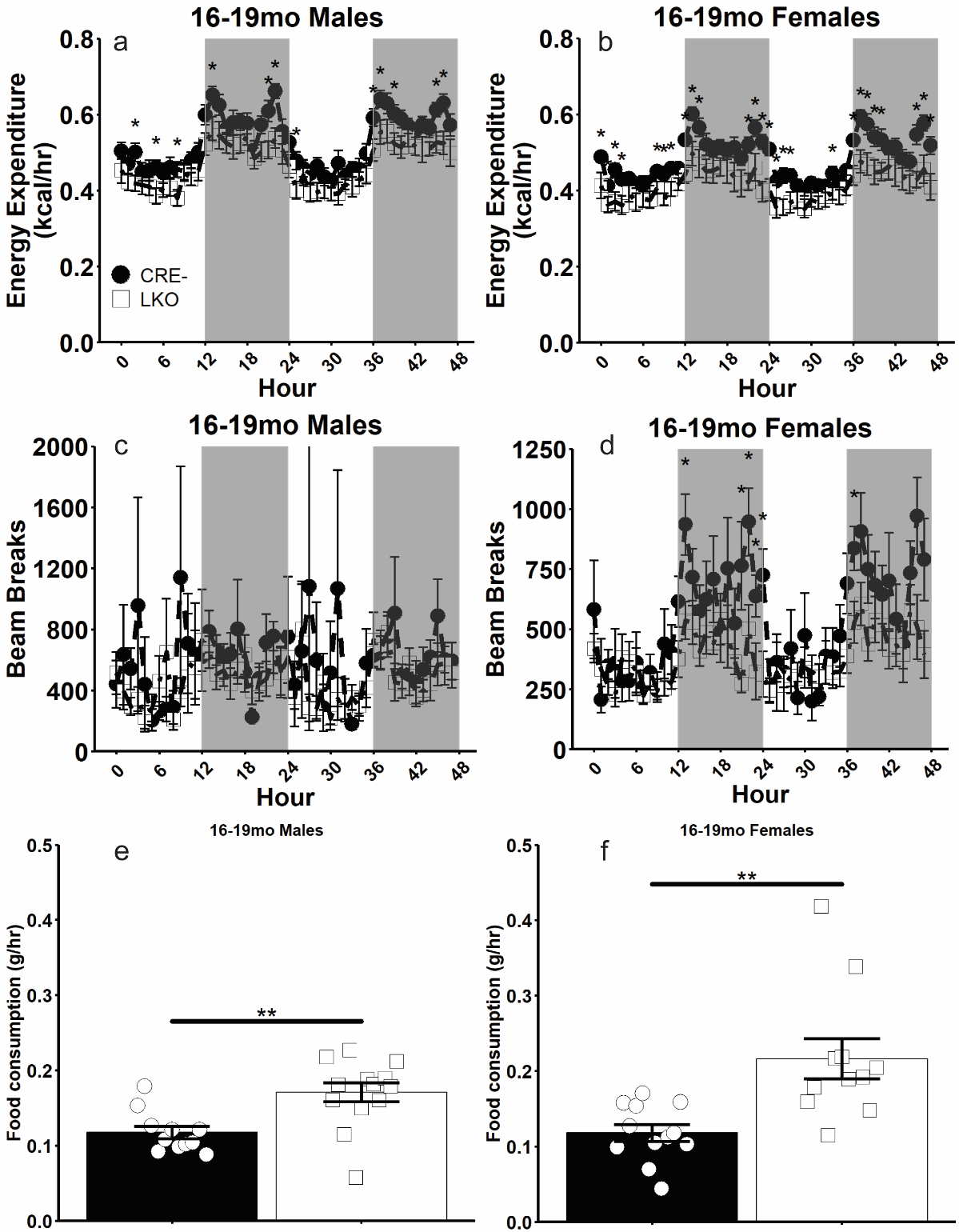


**Supplemental Figure S3**. Bodyweight adjusted energy expenditure in male (a) and female (b) mice calculated during indirect calorimetry. Ambulatory activity, determined by the number of infrared beam breaks recorded for each mouse, calculated during indirect calorimetry for males (c) and females (d). Mean hourly food consumption for male (e) and female (f) mice recorded during indirect calorimetry. *p<0.05, **p<0.01, as determined by two-way ANOVA with Tukey HSD post-hoc comparisons (a-d) or by two-tailed t-test (e-f). Data presented as mean ± SEM with points representing individual mice. N=11-13 per group.


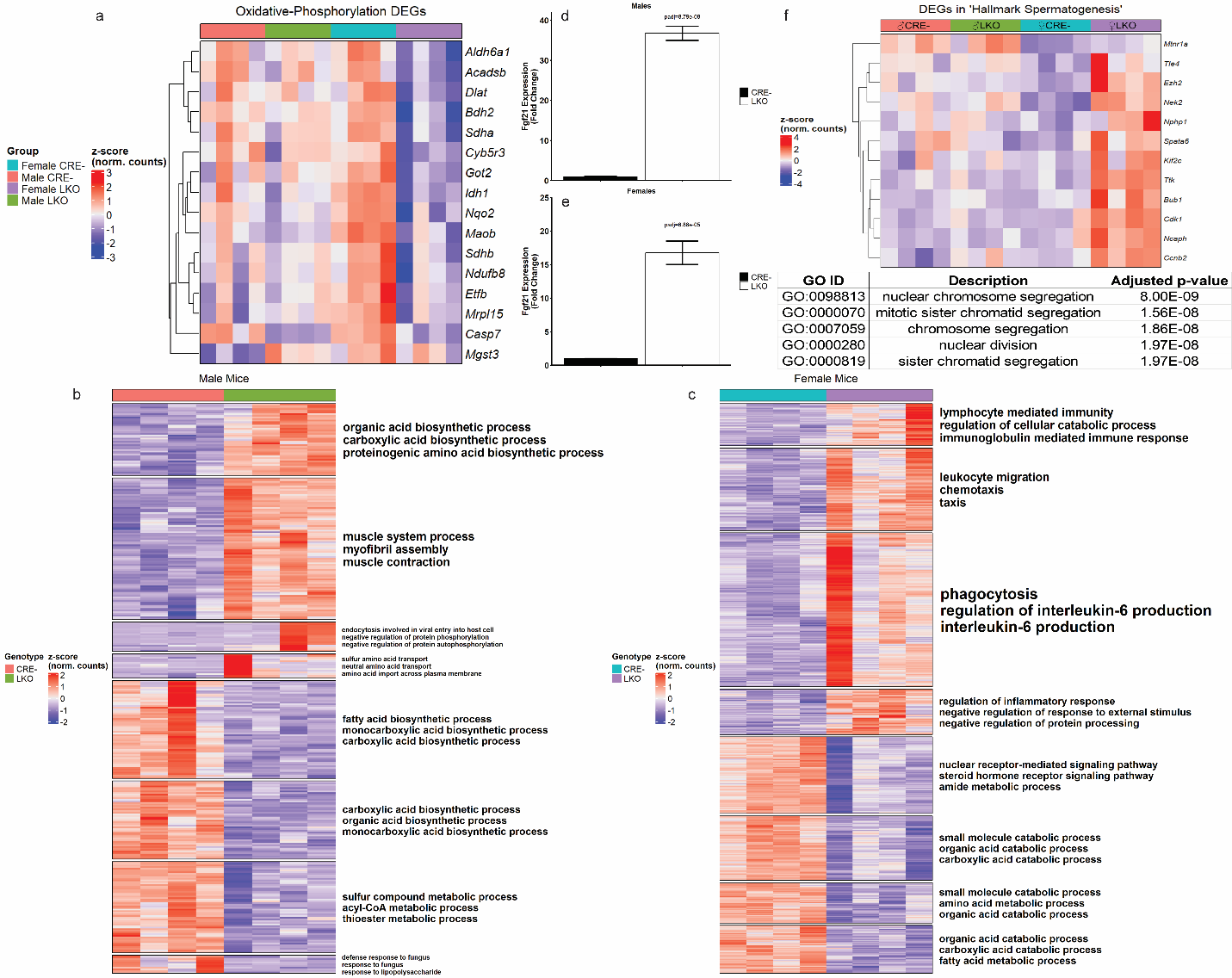


**Supplemental Figure S4.** Heat map visualization of differentially expressed genes (DEGs) in the oxidative-phosphorylation gene set (a). Heat map k-means clustering of DEGs in male LKO mice (b) and female LKO mice (c), relative to sex-matched controls. The top 3 over-represented gene ontology biological process terms associated with each cluster are presented to the right (b-c). Fold change in liver *Fgf21* expression, reproduced from the DESeq2 output with the log2 transformation removed from the fold change values in male (d) and female (e) mice. DEGs in the Hallmark Spermatogenesis gene set, with top 5 over-represented gene ontology biological process terms associated with these genes (f). Padj values represent the adjusted p-values from the DESeq2 output. Color scales represent z-scores of normalized counts. N=4/group.

b

**
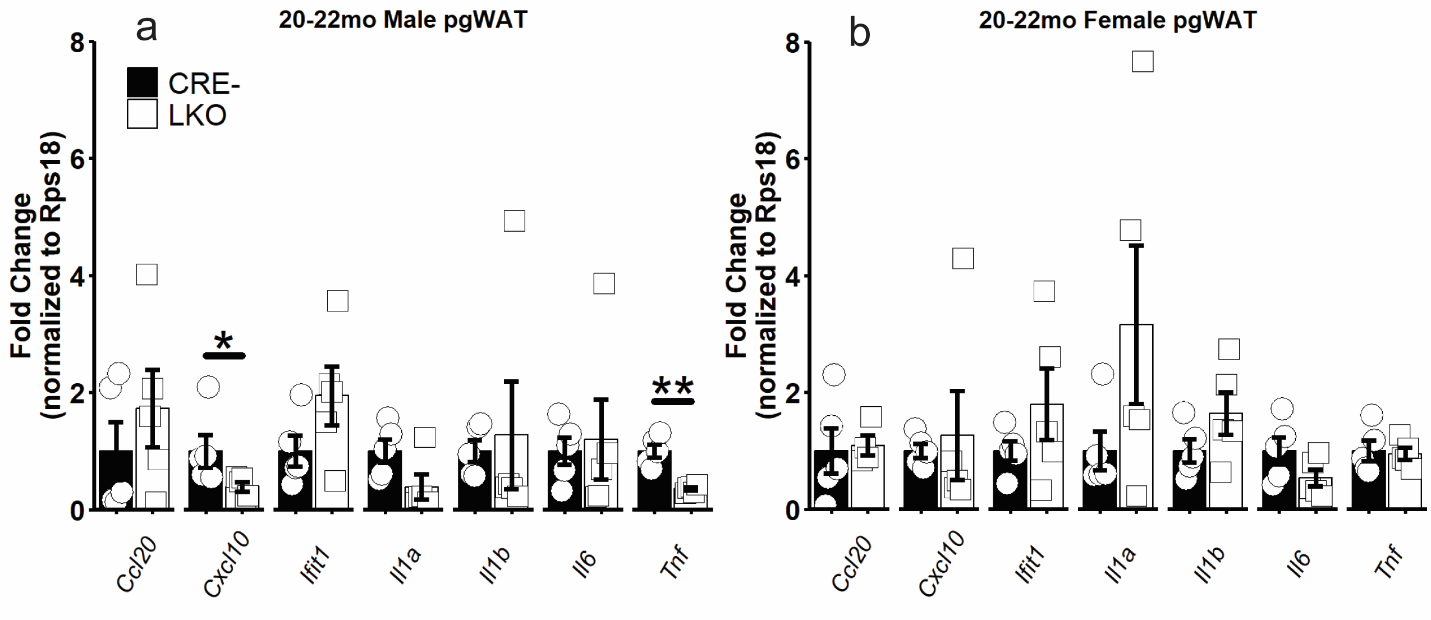
**

**Supplemental Figure S5**. White adipose gene expression. Relative gene expression in perigonadal visceral white adipose tissue (vWAT) in male (a) and female (b) mice with *Rps18* used as an endogenous control. **p<0.01 as determined by two-tailed t-test. Data presented as mean ± SEM with points representing individual mice. N=5 per group.

**Supplemental Tables**

**Supplemental Table 1** Differently expressed genes detected in male livers during RNA-seq data analysis.

**Supplemental Table 2** Differently expressed genes detected in male livers during RNA-seq data analysis.

**Supplemental Table 3** Antibodies used in western blotting experiments

**Supplemental Table 4** Primer sequences used
